# Aim for Clinical Utility, Not Just Predictive Accuracy

**DOI:** 10.1097/EDE.0000000000001173

**Published:** 2020-04-02

**Authors:** Michael C. Sachs, Arvid Sjölander, Erin E. Gabriel

**Affiliations:** From the a Department of Medicine, Solna Eugeniahemmet, T2, Karolinska Universitetssjukhuset, Stockholm, Sweden; b Department of Medical Epidemiology and Biostatistics, Karolinska Institutet, Stockholm, Sweden.

**Keywords:** Predictive signature, Prediction modeling, Emulated trials, Clinical utility

## Abstract

The predictions from an accurate prognostic model can be of great interest to patients and clinicians. When predictions are reported to individuals, they may decide to take action to improve their health or they may simply be comforted by the knowledge. However, if there is a clearly defined space of actions in the clinical context, a formal decision rule based on the prediction has the potential to have a much broader impact. The use of a prediction-based decision rule should be formalized and preferably compared with the standard of care in a randomized trial to assess its clinical utility; however, evidence is needed to motivate such a trial. We outline how observational data can be used to propose a decision rule based on a prognostic prediction model. We then propose a framework for emulating a prediction driven trial to evaluate the clinical utility of a prediction-based decision rule in observational data. A split-sample structure is often feasible and useful to develop the prognostic model, define the decision rule, and evaluate its clinical utility. See video abstract at, http://links.lww.com/EDE/B656.

## Abstract 2

Supplemental Digital Content is available in the text.

Predictions of the prognosis of a patient given their current health state are prolific in medicine, as are the studies and tools to obtain such predictions. For example, there are hundreds of available risk scores for cardiovascular disease,^1^ dozens for prediction of breast cancer occurrence,^2^ dozens for prediction of prostate cancer occurrence and progression,^3^ several prediction models for sepsis in the intensive care unit,^4^ and tens of prediction models of type II diabetes.^5^ Risk scores, risk rankings, clinical outcome predictions, or prognostic predictions, regardless of the name, are all attempting to predict with high accuracy the future outcome of a patient given current information. Despite the ubiquity of such predictions, the vast majority of the predictions and tools go unused in clinical practice.^6^ The lack of use may be due to the lack of actionable information provided by risk scores alone. Physicians want tools to help make treatment decisions, and to achieve this aim, one must clearly specify the intended use of a prognostic model at the outset of the study.

If the intended use of a prognostic model is to drive treatment decisions, a prediction-based decision rule, then it is a medical device, and governing bodies in the United States and Europe have made it clear that evidence of positive impact on patients is needed in order for such devices to be approved for clinical use.^7^ This requires evaluating the clinical utility, i.e., the comparison of expected patient outcomes under use of the prediction-based decision rule to those outcomes had the patients received the standard of care. The gold standard for generating evidence of superior clinical utility is a randomized controlled trial. While well established for the evaluation of treatments, direct randomized comparisons of prediction-based decision rules to standard of care have received less attention.^8^

Very few randomized clinical trials of prediction-based decision rules exist, and even when randomized trials evaluate prediction-based decision rules, they tend to look for an interaction between treatment effect and prognosis rather than directly assessing the clinical utility. For example, the Oncotype DX risk score is currently used to inform whether chemotherapy should be used in addition to hormonal therapy.^9^ Two randomized trials have involved the Oncotype DX risk score^10^ and^11^ and both trials evaluated interactions of the score with randomized treatments rather then directly comparing to the standard of care for treatment selection. This lack of randomized evaluations of clinical utility may be due to misunderstandings about the goals of the risk score or the unwillingness to risk scarce resources on a trial that does not compare randomized treatments.

Our goal is to illustrate how observational data can be used to optimize and evaluate the clinical utility of a prediction-based decision rule. In the context of comparing the effectiveness of treatments, Hernán and Robins^12^ have proposed and promoted the concept of emulating a target trial with observational data, and this concept has also been applied to assess the utility of screening^13^ and in other clinical settings.^14^ In this article, we similarly propose to use observational data to emulate a target trial to assess the clinical utility of a prediction-based decision rule. Our proposal shares some key features with the emulated treatment trial, such as the clear specification of the eligibility, so we will focus on the special considerations and the additional components that are required for the development and evaluation of prediction-based decision rules. By using observational data to optimize and evaluate the clinical utility of a prediction-based decision rule, future confirmatory randomized clinical trials can be better-informed and motivated.

To make things concrete, we will consider the setting of major abdominal surgery in Crohn’s disease. Major abdominal surgery due to Crohn’s disease is considered a serious adverse outcome and is responsible for high healthcare costs and decrease in quality of life in people with Crohn’s disease. Identifying individuals at high risk for surgery may allow for targeted use of early therapeutic interventions to offset this natural course. Several researchers have developed risk prediction models for Crohn’s disease-related surgery and complications.^15,16^ There have been randomized studies to compare the efficacy of early combination therapy to the standard of care,^17^ but there has been no attempt, to our knowledge, to determine whether prediction-based decisions are beneficial. Throughout this article, we will illustrate our concepts using this example. We provide a glossary of terms in the first section of the eAppendix; http://links.lww.com/EDE/B646, followed by a step-by-step description of our proposed approach using this example.

## Developing a Proposal for a Prediction-based Decision Rule

To formalize the optimization of a prognostic prediction in medical decision-making and arrive at a proposal for a prediction-based decision rule for which we can then evaluate the clinical utility, the set of possible decisions needs to be specified. The prediction-based decision rule can then be viewed as a mapping of the prognostic model result to the set of all possible, and reasonable, medical decisions. This set is the action space. The best decision rule, among those that can be evaluated given your data, is obtained by finding the mapping that optimizes the expected clinical utility and minimizes any adverse outcomes or losses. The ideal way to do this is to build a utility function, i.e., a mapping from the decision rule to a weighted set of the possible outcomes, containing all the possible benefits, adverse events, and costs of treatment, and optimize this over a set of groupings based on the prediction and the possible treatments. One potential method for doing this is suggested in Vickers et al,^18^ but there are numerous ways to arrive at a “best” proposal for a prediction-based decision rule.

Suppose we want to use the prediction model for risk of surgery in Crohn’s disease described in Sachs et al^16^ to help determine which treatment patients should receive. As demonstrated in the article, the predictive accuracy of the model is adequate for prognostic prediction alone, but as we will outline, this accuracy may be more or less important based on the how the decision rule is developed. To make the problem feasible, we might only consider different cutoffs of the prognostic prediction for major abdominal surgery in Crohn’s disease to define high and low risk, rather than the full (infinite) space of all possible groupings. Additionally, we will only consider two possible interventions (the action space), to treat with monotherapy (thiopurines alone) or to treat with combination therapy (thiopurines plus biologics). The optimal decision rule could then be any combination of these treatments and the cutoff between high and low risk based on the predictions. Our utility function is simply the proportion of patients who undergo surgery within 5 years, which we want to minimize.

To further reduce the optimization problem, we can separate these two pieces. For example, we could use a predictiveness curve,^19^ which can be used to describe the distribution of the outcome conditional on the prediction, to select the “best” cutoff to define high and low risk. In our example, after the cutoff between high and low risk has been determined, there would only be two possible combinations of therapy and predicted risk: (1) low risk gets thiopurines alone and high risk gets thiopurines plus biologics, or (2) high risk gets thiopurines alone and low risk gets thiopurines plus biologics. One could estimate the utility of both of these decision rules in an observational data set, as we outline in detail in the eAppendix; http://links.lww.com/EDE/B646. However, in practice in our setting, as is often the case, expert opinion dictates that thiopurines plus biologics should not be given to low-risk patients. Although this is not directly optimizing the decision rule, expert opinion still allows us to develop a proposal for the prediction-based decision rule that would be useful in practice.

Regardless of how it is performed, this optimization is something that can be done in the observational setting prior to the running of the clinical trial, and there are strong advantages to doing so. In a clinical trial, only a small set of decision rules can be considered, limiting the ability to optimize the decision. Instead, in the observational setting, all decision rules within the plausible space can be considered and proper utility/loss functions can be used to account for more than just the primary outcome, provided those decision rules are presented in the data. As noted above, the solution to the general problem of whom to treat with what is a much larger problem, with a long literature motivated by subgroup identification in clinical trials. Even when only considering a currently available predictive algorithm and a small set of therapies, the possible approaches to picking the “best” prediction-based decision rule are numerous. The important feature of any useful method of optimization or selection of a proposed prediction-based decision rule is that it be data driven, and therefore it will need to be externally validated. For this, we suggest that a randomly split validation set be used to run the emulated target study as outlined in the next Section.

## Evaluating a Proposed Prediction-based Decision Rule in an Emulated Trial

**FIGURE 1.**
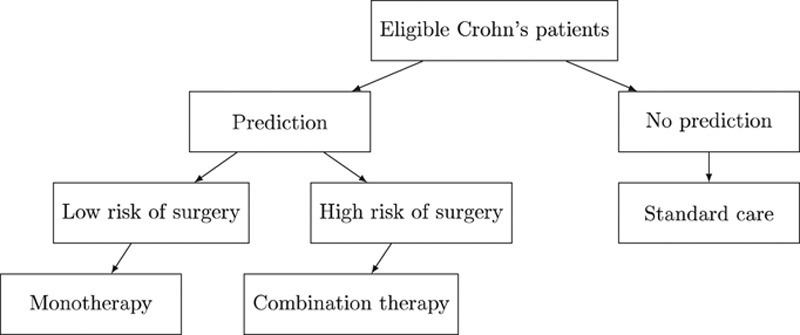
Overview of the target prediction-model based trial in which patients are randomized to a prediction-based treatment decision rule versus standard of care.

Suppose that we have a proposed decision rule for selecting between mono- and combination therapy in patients recently diagnosed with Crohn’s disease, and before this rule is used in clinical practice, we wish to test it in some way for superior clinical utility. The highest quality evidence would be generated by a direct randomized comparison between the prediction-based decision rule and the standard treatment decision. Figure 1 depicts this design for our running example in Crohn’s disease. Note that the only randomization is between using the prediction-based decision rule or the standard of care, which may or may not use other decision rules. The Table summarizes the main components of a hypothetical target trial designed to compare the use of our prediction-based decision rule versus the standard of care.

**TABLE.**
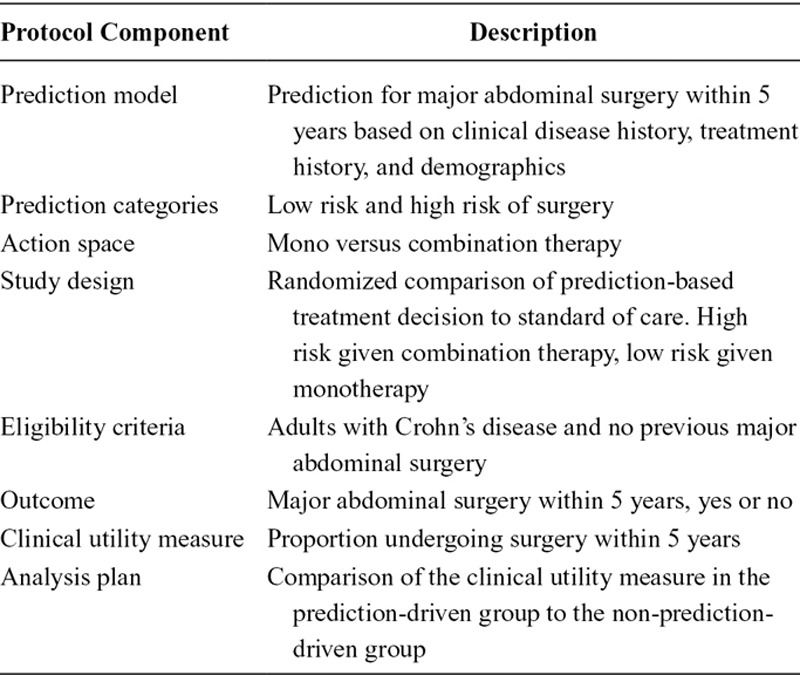
A Summary of a Target Trial to Evaluate the Clinical Utility of a Proposed Prediction-Based Decision Rule in Crohn’s Disease

In this study, we would obtain sufficient data on eligible study participants at the time of Crohn’s disease diagnosis to predict their risk of surgery within 5 years using the fixed prognostic model described above. Then, subjects would be randomized to the prediction-based decision arm, or the non-prediction-based decision arm (standard care). The decision process with the higher expected utility, in this case measured by the proportion undergoing surgery within 5 years, would be more beneficial to use in clinical practice. In the absence of this target randomized trial, we can use a large observational database to provide preliminary evidence or to motivate undertaking a randomized study by emulating this trial.

Hernán and Robins^12^ propose a framework and methods for using causal inference in observational data to emulate a clinical trial for comparing treatments. Just as they describe for the treatment comparison setting, we must define the eligibility criteria for our population of interest that would be used in the target trial, and use similar criteria in our observational emulation. The criteria for our example are given in the Table. The eligibility criteria define our time zero, as subjects would be enrolled as soon as they meet the criteria. Not all subjects will start therapy of any type precisely on the day when the eligibility criteria are met, and failing to account for this time window from eligibility to treatment can potentially impact the feasibility of conducting the study. In addition, to avoid any survivor bias that this grace period may cause, subjects having the event within the grace period should be included in both arms. This concept is illustrated graphically in eFigure 1; http://links.lww.com/EDE/B646 of the eAppendix; http://links.lww.com/EDE/B646.

In our example, time zero would need to be shortly after Crohn’s disease diagnosis, when the initial treatment decision is made. In our emulated study, we would set the grace period to 2 weeks, allowing subjects assigned to mono- or combination therapy to be considered given those therapies if they were assigned them within 2 weeks of Crohn’s disease diagnosis. Subjects having surgery prior to 2 weeks post diagnosis with Crohn’s disease would need to be included in both arms regardless of treatment.

Under certain assumptions, we can estimate the expected potential outcome for subjects using the prediction-based decision rule. However, unlike the trials outlined in [12], the comparison is not between two treatments but between a prediction-based decision rule and the standard of care. The standard of care group will simply be the observed population, using the same eligibility criteria and the same time zero, but will include everyone. All subjects, regardless of what treatment they received, who meet the eligibility criteria will thus be included in the standard of care arm.

## Estimands and Estimation

For simplicity, let 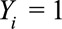 indicate subject *i* having surgery within 5 years of Crohn’s disease diagnosis, and 0 otherwise. Let the two treatment options in our example be labeled *A* for monotherapy and *B* for combination therapy. The comparison of interest in a randomized clinical trial run as in Figure 1 is the proportion of subjects that underwent surgery within 5 years in the arm that used the prediction to determine therapy compared with the proportion of subjects that underwent surgery within 5 years that were randomized to standard of care.

The intention to treat estimand is


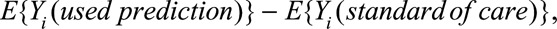


where 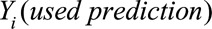 is the potential outcome for subject *i* under use of the prediction, the outcome under the use of prediction regardless of what subject *i* was actually assigned to, and 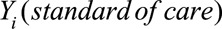 is their potential outcome under standard of care. Under standard assumptions in a randomized clinical trial, 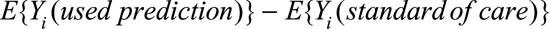 can be unbiasedly estimated by using the observed conditional outcomes 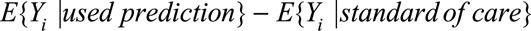, i.e. as the difference in observed proportions between those who were factually assigned to prediction and standard of care, respectively.

In our observational setting, however, we cannot equate 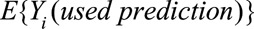 with 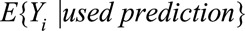 because individuals were not randomized to use the prediction-based decision rule. Instead, we can consider what makes up this expectation, given the deterministic treatment rule. The estimand 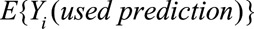 in the trial can be decomposed as


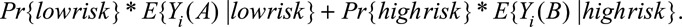


We can easily estimate both 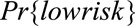 and 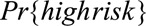 provided our observational sample is a simple random sample of the population of interest. The expectations 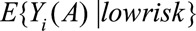 and 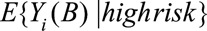 are conditional means of potential outcomes and thus require that we account for any confounders between therapy *A* and the outcome within the low-risk group, as well as any confounders between therapy *B* and the outcome in the high-risk group.

One way to estimate these potential quantities is through g-computation.^20^ Considering 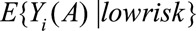 first, subset to those patients classified as low risk. Then specify and estimate a regression model, often referred to as the Q model, for the mean outcome conditional on the observed treatment 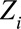 and observed covariates 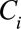 using this subgroup:


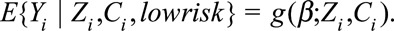


Then, predicted potential outcomes for each subject are obtained by setting 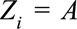 for each subject, combining with their observed covariates and the estimated regression coefficients, and obtaining a prediction 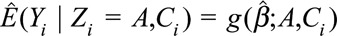. Our estimated mean of the potential outcomes is the average of these predictions. The validity of this estimate relies on several assumptions:

1. Positivity of treatment assignment: Within each subgroup defined by the covariates 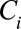, there must be a positive probability of receiving treatment *A*.
2. No unmeasured confounding. All confounders of the effect of treatment on the outcome are measured.
3. Correct specification of the Q model. The model above is correctly specified in terms of the treatment and covariates.

Under these assumptions, our suggested estimated potential outcome means are consistent for the true potential outcome means. The argument applies equally to the estimation of 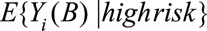 and other similar quantities. The data needed to fulfill these assumptions will clearly vary with the treatments and outcomes of interest. However, the data must contain subjects receiving all treatment types of interest in each of the risk groups, and must have all confounders measured and observed.

The final piece of the desired estimand, 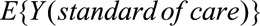, is easier to estimate and requires no additional assumptions. As all subjects in the observational data set that meet the eligibility criteria received the standard of care, the potential outcome is the factual outcome so this can simply be estimated by calculating the proportion of subjects that underwent surgery within 5 years of diagnosis, 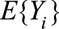.

We are glossing over some of the details regarding estimation in these cases, and thus we will not outline how inference should be undertaken, in general. However, it should be noted that although the subjects contributing to the estimation of 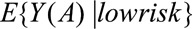 and 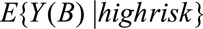 will be different, all subjects will contribute to the estimation of 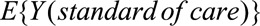, and this must be accounted for in the variance estimate of the final comparison, for example, with the nonparametric bootstrap. In the eAppendix; http://links.lww.com/EDE/B646, we work through a step-by-step example of our proposed framework to demonstrate how it works.

**FIGURE 2.**
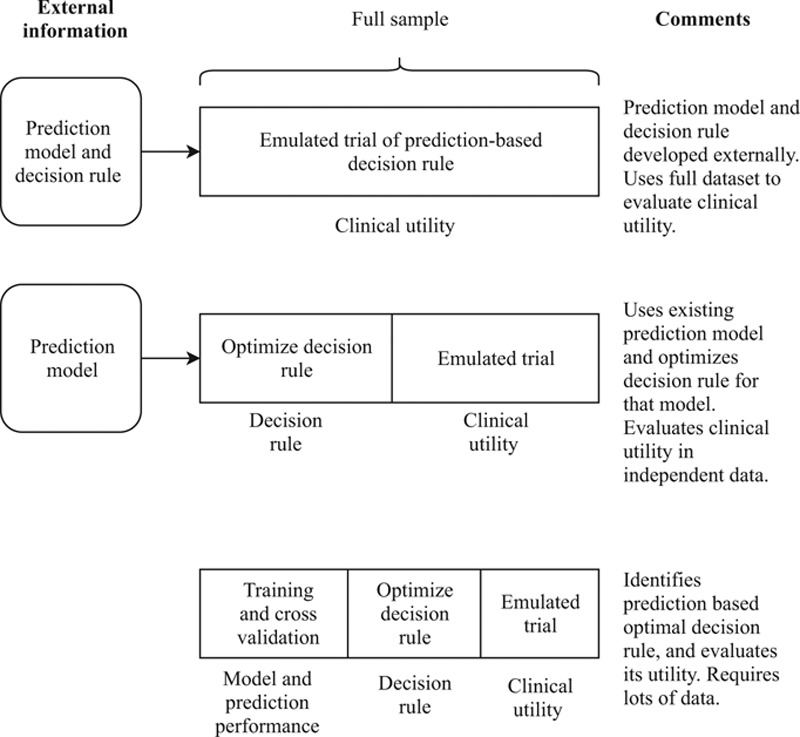
Overview of strategies for split sample prediction model training and clinical utility assessment in observational settings.

We have outlined our conceptual framework for developing a proposed prediction-based decision rule and evaluating its clinical utility in an emulated clinical trial. One can view this development and evaluation process of a prediction-based decision rule in three separate steps: first developing a prognostic model, then determining a proposed decision rule based on it, and finally evaluating the proposed decision rule for superior clinical utility. In a data-rich scenario, such as a large population based disease register, it may be possible to randomly split the dataset into a training sample, optimization sample, and clinical assessment validation sample. Figure 2 illustrates the possible strategies for split-sample training and optimization, and comments on their merits. If a good prognostic prediction model has already been developed, then it can be considered external information and only a two-way data split would be needed to do the second and third step. Likewise, if the prediction-based decision rule has already been proposed externally, all of the data can be used for the third step. In less data-rich scenarios, it may be possible to use cross-validation or bootstrapping techniques to make more efficient use of available data for the development of a prediction model and optimization of the decision rule. As always, the bootstrap can be used in the prediction step to obtain, potentially better, estimates of the prediction error.^21^ The emulated trial, however, should aim to evaluate the clinical utility of a specific, fixed decision rule, in an independent sample.

## DISCUSSION

If a prognostic prediction will be used to drive medical decisions, that prediction-based decision rule should be optimized and tested independently to determine whether it is worthwhile. To optimize and test a prediction-based decision rule, one must first define the space of possible actions that can be taken at the time or shortly after the prediction is made and propose a deterministic rule based on the predictions optimally. Then, evidence must be generated that the use of the decision rule based on the prediction model leads to improved clinical outcomes in comparison to the current standard of care. Often, the optimization in the development phase is overlooked, or is based on expert opinion without formal optimization, while the direct assessment of the utility of the prediction-based decision rule in comparison to the standard of care is even more rare.

We have outlined one approach to the development, optimization and evaluation of a prediction-based decision rule. This approach involves the optimization of a proposal for a prediction-based decision rule in observational data and the specification of the target prediction driven trial of interest, which can then be emulated in independent observational data, to formally evaluate the clinical utility of the optimized prediction-based decision rule.

The ideal procedure to develop an optimal decision rule based on baseline information may not involve prognostic prediction at all. Instead, one could directly optimize a decision rule for a utility function that quantifies all the benefits in terms of the relevant clinical outcomes, treatment decisions, as well as the costs. This decision rule would be a direct function of all available baseline information at the point a treatment decision was needed. There has been previous work on this in the context of identifying biomarkers for treatment selection in randomized trials.^22,23^ This may be practically infeasible, due to limitations on the observed treatments under study, and computationally challenging due to the high dimensional space in which one needs to optimize.

Using our proposed framework in observational data allows for optimization and assessment and therefore has the potential to improve current medical practice in a timely manner. Clearly, a randomized clinical trial evaluating a prediction-based decision rule would be ideal. In lieu of this gold standard, an emulated trial can be used. As with all observational studies aiming to estimate causal contrasts, confounding is a major limitation. Prediction-based decision rules imply some special considerations regarding confounding. Prediction models are generally not designed with consideration of causal relationships, so colliders may be included in the models—see Sjölander^24^—and this has implications for the nature of the confounding that one needs to consider and control for. The precise conditions under which this is possible for different estimators needs to be explicitly investigated, and, like the assumption of no unmeasured confounding, it is likely that the assumptions that generate these conditions are untestable. However, quantitative bias analysis^25,26^ is a sensible approach to assessing the sensitivity of one’s findings to these assumptions that should be further developed in this context.

One practical limitation of our suggested approach is that the set of actions under consideration need to be observed in the sample to a sufficient degree to estimate the relevant conditional expectations (the positivity assumption). This may not be possible if the action space contains novel or unapproved treatments. For example, decision rules to treat with novel cancer drugs cannot be assessed unless those drugs are administered in practice. Other practical considerations, such as survivor and selection biases, which are inherent in retrospective studies, can be reduced by carefully planning studies with pre-specified protocols that mimic those used in randomized controlled trials.^12,27^ This approach has been suggested and applied in comparative effectiveness research^13,14,28^ and we advocate for a similar approach.

The optimization and utility assessment of a decision rule may depend on more than simply the clinical outcome. For instance, cost and quality of life considerations are important in many settings. Our proposed framework can apply such a utility function in both the optimization and evaluation phases, as long as the utility function is pre-specified and estimable using observed data. This is a concept that should be considered even in randomized clinical trials, but is likely easier to apply in register data, where many more measurements are available.

We have outlined the use of one target trial design for the evaluation of prediction-based decision rules, but as summarized in Micheel et al,^29^ Chapter 4, there are a variety of study designs that can be used to assess the value of predictions for guiding treatment decisions. Elucidation of this framework in alternative trial designs as well as efficient use of available data for the optimization step are of future research interest for the authors, as are applications of these methods in real data settings.

## Supplementary Material


