# Supplementary material for: Aim for Clinical Utility, Not Just Predictive Accuracy
Source: Epidemiology. 2020 Apr 2;31(3):359–64. doi: 10.1097/EDE.0000000000001173 (PMC7144757; doi:10.1097/EDE.0000000000001173)
Supplement: Supplementary file 1 [file ede-31-359-s001.pdf]

Aim for clinical utility, not just predictive accuracy:

Supplementary eAppendix

Michael C Sachs   Arvid Sjölander   Erin E Gabriel

November 13, 2019

M.C. Sachs: Department of Medicine, Solna

Eugeniahemmet, T2, Karolinska Universitetssjukhuset,

171 76 Stockholm, Sweden

Tel.: +46 08 517 761 42

michael.sachs@ki.se

A. Sjölander and E.E. Gabriel:

Department of Medical Epidemiology and Biostatistics,

Karolinska Institutet,

171 65 Stockholm, Sweden

Glossary of terminology

Prognostic prediction A mathematical combination of multiple covariates/measurements that is used to calculate probabilities (or scores on other scales) of future events for individuals [1].

Action space The set of possible decisions under consideration in a clinical context (e.g. to treat or not to treat) [2].

Utility function A mathematical quantification of the benefits, costs, and risks associated with decisions and future outcomes.

Prediction-based decision rule A specified, logical and reproducible method for taking a prognostic prediction as input, and determining an element from the action space.

Clinical utility The comparison of the expected clinical outcome of the population of interest under the use of the prediction-based decision rule to the expected outcome under the standard of care.

Step-by-step Guide

To make things concrete, we will consider the setting of major abdominal surgery in Crohn's Disease (CD). Major abdominal surgery due to CD is considered a serious adverse outcome, and is responsible for high health care costs and decrease in quality of life in people with CD. Identifying individuals at high risk for surgery may allow for targeted use of early therapeutic interventions to offset this natural course. We have simulated data to mimic a large national cohort of CD patients with clinical information, demographics, treatment history, and up to 10 years of follow up. Here we outline the steps that we will undertake to develop and evaluate a prediction based decision rule.

Preparation and planning

1. Decide on eligibility criteria for the potential randomized clinical trial to assess a prediction based decision rule. This includes both patient characteristics and the time at which the decision will be made, e.g., within 2 weeks of diagnosis. Record this information in a draft protocol.
2. Assemble a cohort of patients who meet the eligibility criteria. Define covariate data that is available before the time at which the decision is to be made, and then the clinical outcome of interest and the treatments that are observed at the time when the decision is to be made. Record this information in the draft protocol.
3. Randomly allocate patients in the cohort into 3 groups: Model, decision rule, and clinical utility. If possible, keep the second group completely separate from your working environment until Development of decision rule and the third group separate until step Evaluation of the decision rule.

Development of prognostic model

1. In the first model subcohort, decide on the statistical techniques that will be used to develop prediction models. Record this information in a protocol.
2. Apply the statistical techniques to develop a prediction model for the clinical outcome using the covariates that are available before the decision.
3. In a cross-validation framework, assess the accuracy of the prediction model development process that was used in the previous step.
4. Report the prediction model algorithm and its cross-validated performance. Test that the algorithm is reproducible and can be applied to new data in the same format.

Development of decision rule

1. In the decision rule subcohort, apply the prediction algorithm developed in the previous step. Examine the distribution of the predictions in this new cohort. Use predictiveness curves and clinical consultation to decide on cutoffs for high risk versus low risk. Record this decision making process and the results in a protocol.

2. In consultation with an expert on the clinical context, and in view of the observed treatments in the sample, decide what the action space is. Record this in a draft protocol.

3. Decide on the utility function that measures the utility of the treatment assignment. This is a function of the clinical outcome at a minimum, but may also incorporate quantifications of costs and risks associated with specific treatments. Record this in the protocol.

4. Estimate the average utility for each combination of treatments in the action space and risk groups, accounting for confounders of the treatment outcome association using g-computation. The treatment with the highest average utility in each risk group defines the proposed prediction-based decision rule. Record this decision rule in the protocol.

Evaluation of the decision rule

1. In the clinical utility cohort, apply the prediction algorithm and cutoffs to obtain the classification into high risk and low risk.

2. Specify the grace period as the time from eligibility to when a treatment was received (e.g., two weeks), and use this to define the observed treatment for each individual in the sample. See eFigure 1 below.

3. In the subgroups of individuals classified as high and low risk separately, specify and fit a regression models for the outcome as a function of the observed treatment and observed confounders of the treatment-outcome association. Make sure to include relevant treatment-covariate interactions.

4. Using the models estimated in step 3, obtain predictions for each subject by risk group using their observed covariates, and setting their treatment to the treatment prescribed by the proposed decision rule. These are the predicted potential outcomes.

5. Term 1 of the estimand is the mean of the predicted potential outcomes times the proportion classified into each risk group and then added together:

$$M_1 = Pr\{\text{low risk}\} * E\{\hat{Y}_i(A)|\text{low risk}\} + Pr\{\text{high risk}\} * E\{\hat{Y}_i(B)|\text{high risk}\}.$$

6. Term 2 of the estimand is the mean outcome in all subjects: $M_2 = E\{Y_i\}$.

7. The estimated clinical utility is $M_1 - M_2$.

8. To estimate the standard error, take a large number nonparametric bootstrap samples, and repeat steps 3-7 for each sample. The distribution of bootstrapped estimates can be used to calculate confidence intervals for the clinical utility.

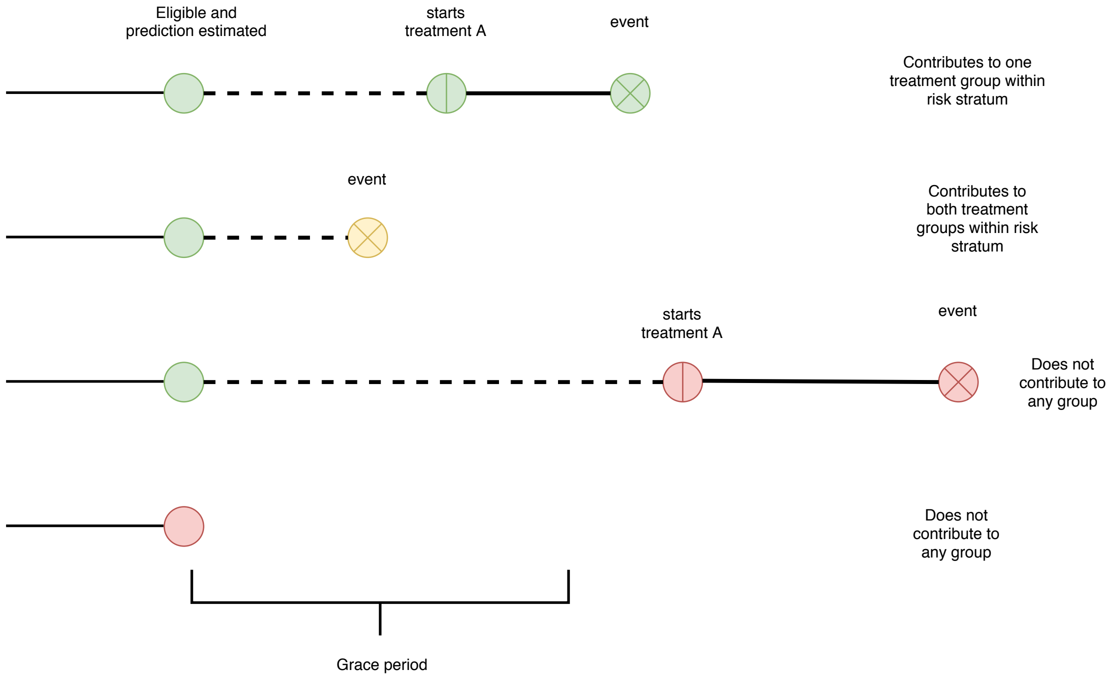

eFigure 1: Illustration of eligibility criteria, treatment initiation, and the grace period.

R code with a simulated example

```
set.seed(20190826)

samp_data <- function(n = 2000) {

    X <- matrix(rnorm(n * 3), ncol = 3)
    trt.A <- rbinom(n, 1, p = pnorm(X[, 1] * 1 + X[, 3] * 2))

    risk.p <- pnorm(X[, 1] * .5 + X[, 2] * 1 + X[, 3] * .75)
    highrisk <- risk.p > .75

    true.Risk <- rowSums(X)
    q3 <- quantile(true.Risk, 0.75)
    # potential outcomes: 1  = get trt.A if low risk,
    #                     0  = randomly assign trt.A according to X[, 1] and X[, 3]
    #                    in truth, trt.B is effective only at high risk



    p0 <- pnorm(true.Risk - ifelse(true.Risk > q3, 2*(1 - trt.A), 0))
    Y0 <- rbinom(n, 1, p = p0)


    trt.A.star <- as.numeric(!highrisk)
    p1 <- pnorm(true.Risk - ifelse(true.Risk > q3, 2*(1 - trt.A.star), 0))

    Y1 <- rbinom(n, 1, p = p1)

    data.frame(X, trt.A, highrisk, Y = Y0, Y1 = Y1)

}

estimate_utility <- function(cucohort) {
    ## Step 3

    fit.high <- glm(Y ~ (X1 + X2 + X3) * trt.A, family = "binomial",
                    data = subset(cucohort, highrisk == TRUE))
    fit.low <- glm(Y ~ (X1 + X2 + X3) * trt.A, family = "binomial",
                   data = subset(cucohort, highrisk == FALSE))

    ## Step 4
    ## low risk gets treatment A

    newlow <- subset(cucohort, highrisk == FALSE)
    newlow$trt.A <- 1
    Yhat.A <- predict(fit.low, newdata = newlow, type = "response")

    newhigh <- subset(cucohort, highrisk == TRUE)
    newhigh$trt.A <- 0
```

```r
    Yhat.B <- predict(fit.high, newdata = newhigh, type = "response")

    ## Step 5

    M1 <- mean(cucohort$highrisk == FALSE) * mean(Yhat.A) +
        mean(cucohort$highrisk == TRUE) * mean(Yhat.B)

    ## Step 6
    M2 <- mean(cucohort$Y)

    ## Step 7
    M1 - M2
}


data <- samp_data()
mainest <- estimate_utility(data)

## Step 8
bootests <- rep(NA, 1000)
for(i in 1:length(bootests)){
    sampdex <- sample(1:nrow(data), nrow(data), replace = TRUE)

    bootests[i] <- estimate_utility(data[sampdex, ])
}

CI <- quantile(bootests, c(.025, 0.975))
```

The estimated clinical utility of the decision rule is -0.11 95% CI: -0.17 to -0.05. This is interpreted as the estimated difference in the proportion having the outcome comparing the prediction based decision rule arm to the standard of care.

References

[1] Steyerberg EW, Moons KGM, van der Windt DA, et al. Prognosis Research Strategy (PROGRESS) 3: Prognostic Model Research. *PLoS Medicine*. 2013;10(2). ISSN 1549-1277.

[2] Morita S, Müller P. Bayesian population finding with biomarkers in a randomized clinical trial. *Biometrics*. 2017;73(4):1355–1365.
